# *Notes from the Field*: Early Identification of the SARS-CoV-2 Omicron BA.2.86 Variant by the Traveler-Based Genomic Surveillance Program — Dulles International Airport, August 2023

**DOI:** 10.15585/mmwr.mm7243a3

**Published:** 2023-10-27

**Authors:** Stephen M. Bart, Andrew P. Rothstein, Casandra W. Philipson, Teresa C. Smith, Birgitte B. Simen, Azaibi Tamin, Lydia J. Atherton, Jennifer L. Harcourt, Allison Taylor Walker, Daniel C. Payne, Ezra T. Ernst, Robert C. Morfino, Ian Ruskey, Cindy R. Friedman

**Affiliations:** ^1^Division of Global Migration Health, National Center for Emerging and Zoonotic Infectious Diseases, CDC; ^2^Ginkgo Bioworks, Inc., Boston, Massachusetts; ^3^Coronavirus and Other Respiratory Viruses Division, National Center for Immunization and Respiratory Diseases, CDC; ^4^XpresCheck, XWELL, New York, New York.

During August 13–14, 2023, a new SARS-CoV-2 Omicron subvariant with a large number of mutations compared with previously circulating BA.2 variants (>30 amino acid differences in its spike protein) was identified by genomic sequencing in Denmark and Israel and subsequently designated BA.2.86 (*1*,*2*). Given near-simultaneous detections in multiple countries, including the United States, further information was needed regarding geographic spread of BA.2.86. Since January 2022, submissions to SARS-CoV-2 sequence repositories have declined by 95%,* substantially decreasing global capacity to monitor new variants. To fill gaps in global surveillance, CDC’s Traveler-based Genomic Surveillance (TGS) program was developed to provide early warning of new variants entering the United States by collecting samples from arriving international travelers (*3*). 

## Investigation and Outcomes

The TGS program anonymously collects two nasal swab samples from consenting international travelers arriving at six major U.S. airports.^†^ Participants complete a brief questionnaire that collects information including travel history, COVID-19 vaccination status, and previous COVID-19 history. One sample collected from each traveler is pooled together with up to nine other travelers’ samples and tested for SARS-CoV-2 using reverse transcription–polymerase chain reaction. If a pooled sample tests positive, it undergoes viral genomic sequencing. The second nasal samples from each traveler in that pool are then tested for SARS-CoV-2, and positive individual samples are sequenced. Select positive individual samples are sent to CDC laboratories for virus isolation and characterization. This activity was reviewed by CDC, deemed not research, and was conducted consistent with applicable federal law and CDC policy.^§^

On August 17, 2023, genomic sequencing identified BA.2.86 in a pooled sample of swabs from 10 TGS participants collected on August 10 at Dulles International Airport near the District of Columbia. Testing and sequencing of the individual samples confirmed the presence of BA.2.86 in one individual sample on August 20. The sample was collected from a U.S. resident returning from a 15–30-day trip to Japan; health authorities in Japan were notified upon confirmation. The traveler reported no previous COVID-19 infection and had last received a COVID-19 vaccine dose in October 2022. This sequence was the second publicly reported BA.2.86 sequence in the United States and the seventh reported globally,^¶^ preceding the first BA.2.86 sequence submission reported from Japan by 17 days.** The TGS sample was sent to a CDC laboratory, but virus isolation was not successful. Phylogenetic analyses indicated that the TGS sample contained distinct genetic differences from other BA.2.86 sequences collected in August 2023 (Figure), consistent with BA.2.86 circulation and divergence from BA.2.86.1 viruses several months before detection (*4*,*5*).

**FIGURE:**
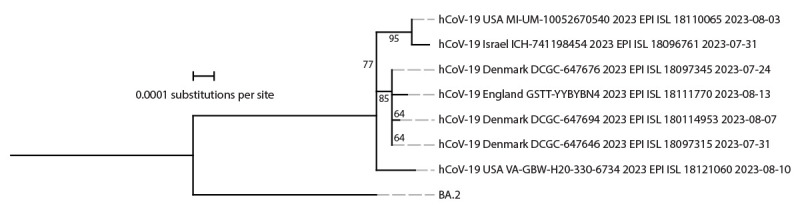
Phylogeny of SARS-CoV-2 Omicron BA.2.86 samples available on Global Initiative on Sharing All Influenza Data* as of August 21, 2023 (seven genomes) and ancestral BA.2 sequences^†^^,^^§^^,^^¶^^,^^**^ — worldwide, August 2023 **Abbreviation:** GISAID = Global Initiative on Sharing All Influenza Data. * https://gisaid.org ^†^ Consensus genome sequences from BA.2.86 GISAID submissions on or before August 21, 2023, were aligned, and mutational profiles were generated using Nextclade (version 2.14.1; https://joss.theoj.org/papers/10.21105/joss.03773). Consensus reference genomes for BA.2 were available at https://github.com/corneliusroemer/ncov-simplest/tree/main/data. A maximum likelihood phylogenetic tree was generated using iqTREE software (version 1.6.12) with 1,000 bootstraps. Using the iqTREE model finder tool, the HKY+F+I model was selected as the most appropriate model according to Bayesian Information Criterion. The maximum likelihood tree was visualized and annotated using iTOL (version 6; https://academic.oup.com/nar/article/49/W1/W293/6246398). Branch labels indicate confidence in phylogenetic placement as a percentage. ^§^ https://academic.oup.com/bioinformatics/article/34/23/4121/5001388 ^¶^ https://academic.oup.com/mbe/article/32/1/268/2925592 ** The BA.2.86 sample identified through the Traveler-based Genomic Surveillance program (hCoV-19 USA VA-GBW-H20-330-6734 2023 EPI ISL 18121060 2023-08-10) was collected at Dulles International Airport on August 10, 2023.

## Preliminary Conclusions and Actions

As a component of comprehensive U.S. SARS-CoV-2 genomic surveillance, TGS detected the BA.2.86 variant within days of its first identification globally, highlighting its importance for the detection of variants entering the United States. This identification provided important context regarding BA.2.86 geographic spread and diversity. Although virus isolation was not successful in this case, continued surveillance and sample collection are important to enable rapid laboratory characterization of variant sensitivity to antibody neutralization and antiviral drugs. Early variant detection among travelers and laboratory characterization of new variants are essential components of CDC’s respiratory illness surveillance, especially as global sequencing volumes decline.

## Footnotes

*
https://gisaid.org/hcov-19-variants-dashboard/

† Newark Liberty International Airport, Newark, New Jersey; John F. Kennedy International Airport, New York, New York; Dulles International Airport, near the District of Columbia; Seattle-Tacoma International Airport, Seattle, Washington; San Francisco International Airport, San Francisco, California; and Los Angeles International Airport, Los Angeles, California. https://wwwnc.cdc.gov/travel/page/travel-genomic-surveillance

§ 45 C.F.R. part 46.102(l)(2), 21 C.F.R. part 56; 42 U.S.C. Sect. 241(d); 5 U.S.C. Sect. 552a; 44 U.S.C. Sect. 3501 et seq.

¶ The TGS sequence was submitted to the Global Initiative on Sharing All Influenza Data (GISAID) repository on August 21, 2023 (accession number EPI_ISL_18121060), and raw sequencing reads were submitted to the National Center for Biotechnology Information Sequence Read Archive on August 23, 2023 (accession number SRX21474168).

** A BA.2.86 sequence from Japan (sample collection date August 24, 2023) was submitted to GISAID on September 7, 2023 (accession number EPI_ISL_18233521). This sequence had mutations that differed from the TGS sample, and unlike the TGS sample, this sequence was assigned to the BA.2.86.1 sublineage.
